# A theory-based evaluation of a dissemination intervention to improve childcare cooks’ intentions to implement nutritional guidelines on their menus

**DOI:** 10.1186/s13012-016-0474-7

**Published:** 2016-07-25

**Authors:** Sze Lin Yoong, Jannah Jones, Josephine Marshall, John Wiggers, Kirsty Seward, Meghan Finch, Alison Fielding, Luke Wolfenden

**Affiliations:** 1 Hunter New England Population Health, Wallsend, New South Wales 2287 Australia; 2 School of Medicine and Public Health, University of Newcastle, Callaghan, New South Wales 2308 Australia; 3 Hunter Medical Research Institute, Newcastle, New South Wales 2300 Australia; 4 Priority Research Centre for Health Behaviour, University of Newcastle, Callaghan, New South Wales 2308 Australia

**Keywords:** Dissemination, Guideline, Childcare services, Nutrition

## Abstract

**Background:**

Childcare services represent a key setting to implement nutritional interventions to support the development of healthy eating behaviours in young children. Childcare-specific nutritional guidelines outlining recommendations for provision of food in care have been developed. Despite this, research suggests that few childcare services currently implement these guidelines. This study aimed to examine the impact of providing printed educational materials on childcare service cooks’ intentions to use nutritional guidelines and provide fruit and vegetables on their menu.

**Findings:**

A randomised controlled trial was conducted with 77 childcare services (38 control and 39 intervention). Intervention service cooks were mailed a two-page educational material together with a menu planning checklist. Intervention development and evaluation was guided by the theory of planned behaviour. Outcome data assessing intentions to use nutritional guidelines and serves of fruit and vegetables provided on menus (primary outcomes) as well as secondary outcomes (attitudes, behavioural regulation and social norms) were collected via a telephone interview with cooks. Relative to the comparison group, cooks in the intervention arm had significantly higher intentions to use the guidelines (*p* value 0.0005), accompanied by significant changes in perceived behavioural control (*p* value 0.0008) and attitudes (*p* value 0.0071). No significant difference in serves of fruit (*p* value 0.7278) and vegetables (*p* value 0.0573) was observed.

**Conclusions:**

The use of educational materials can improve childcare service cooks’ intentions to use nutritional guidelines; however, as a standalone strategy, it may not improve provision of food on menus.

**Electronic supplementary material:**

The online version of this article (doi:10.1186/s13012-016-0474-7) contains supplementary material, which is available to authorized users.

## Background

Poor nutrition is a leading risk factor for the development of chronic diseases including cardiovascular disease, type 2 diabetes and some cancers [1]. Interventions to improve children’s diet are recommended as a strategy to reduce the burden from dietary risk factors, as dietary behaviours established in young children track into adulthood [2].

Childcare services represent a key setting to implement healthy eating interventions as they provide broad reach to young children in the population [3]. Systematic reviews report that environmental and educational interventions to improve childcare provision of food and/or healthy eating curriculum can improve child dietary intake [4]. Internationally, countries including Australia have introduced nutritional guidelines to support provision of healthier foods in childcare services [5, 6]. Despite this, research suggests that few childcare providers adhere to these guidelines [7].

Research suggests that a lack of knowledge of dietary guidelines as well as childcare staff attitudes and perceptions towards the provision of food may represent barriers to provision of foods consistent with guidelines [8]. Education resources [9] may represent a useful strategy to overcome such barriers to guideline implementation. Systematic review and randomised controlled trial evidence describing the effectiveness of education resources in changing clinician guideline implementation has reported that such interventions have a limited impact on changing provider intentions and behaviour [10–14]. These reviews, however, noted limitations such as insufficient sample sizes and inappropriate analyses [12]. A recent Cochrane systematic review which presented findings from 14 randomised controlled trials found that printed educational material had a small effect on physician practice (including guideline implementation) when compared to no intervention [15].

Given the potential of educational materials to make a contribution to improving the menus of childcare services at scale, this study aimed to examine the impact of providing printed educational materials on childcare service cooks’ intentions to use nutritional guidelines and provide fruit and vegetables on their menu. As a secondary outcome, attitudes, perceived behavioural regulation and social norms was also assessed.

## Methods

Ethical approval was provided by Hunter New England and the University of Newcastle Human Research Ethics Committee (HREC). This trial was prospectively registered (ACTRN12615000712505).

### Context

In the state of New South Wales (NSW), Australia, childcare accreditation standards require that services provide foods consistent with the 2013 Australian dietary guidelines [16]. To support this, the state government released the Caring for Children resource (2014), which outlines the recommended number of serves of food groups that childcare services should provide [5].

### Design and setting

This study was a parallel group randomised controlled trial, with post-intervention data collection only. Long day care services (centre-based services typically open ≥8 hours/day) located within NSW, Australia, served as the sampling frame. Services were excluded if they did not undertake menu planning on site or where cooks did not understand English sufficiently to complete the survey.

### Recruitment

An information statement was sent to service cooks 2 weeks prior to being called and invited to participate in a computer assisted telephone interview (CATI).

### Randomisation and allocation

Childcare services were randomly allocated to either the intervention or control condition by a blinded research assistant using a random number function in Microsoft Excel in a 1:1 ratio.

### Intervention group

Approximately 6 weeks prior to being sent the information statement, intervention cooks were mailed a two-page education resource and the menu planning checklist from the Caring for Children resource [5]. The two-page educational material was developed by a local health promotion team consisting of dietitians, behavioural scientists and health promotion practitioners. Consistent with evidence for the development of educational materials, the resource included coloured visuals outlining recommended serve sizes, had endorsement from a reputable health promotion organisation and targeted a specific behaviour (menu planning) [15]. The content of the material was guided by the theory of planned behaviour (TPB). Accordingly, the material was designed to address behavioural, social and control beliefs identified as impeding the use of guidelines in menu planning and food provision [17]. Based on formative work undertaken in the childcare setting, key beliefs were identified and strategies to address each applied to the resource (Additional file 1) [17].

### Control group

The control group received usual care. All services could access the Caring for Children resource online [5] and may have been offered support from their local health promotion staff.

### Data collection and measures

Outcomes were assessed between July and September 2015 via CATI, administered according to a standard protocol by trained interviewers who were blind to group allocation.

#### Cooks and service characteristics

Cooks were asked to report on their age, sex, nutrition qualifications, number of years they had been employed as a service cook and the number of hours worked. Service postcode was obtained from centralised records obtained from a state government agency.

#### Trial outcomes

A 21-item questionnaire, adapted from previously validated tools, assessing intentions (four items), attitudes (five items), social norms (six items) and perceived behavioural control (six items) was used to assess the constructs from the TPB [18] (Additional file 2). The items were scored on a seven-point Likert scale ranging from strongly disagree to strongly agree. Mean values were generated to create a single composite score for all TPB constructs (post-test Cronbach’s alpha for intentions 0.79; social norms 0.73; perceived behavioural control 0.73). Cooks reported using a single question, the number of serves of fruit and vegetables provided on their menu in the last week. Cooks were asked to refer to their menu while reporting.

#### Awareness of the guidelines and other implementation support

Cooks were asked if they had received a copy of the Caring for Children resource and whether they had received any implementation support in the past 6 months.

#### Intervention receipt

Cooks reported whether they recalled receiving the educational material and if so how long ago they received it. Those who reported receiving the material were asked if they had used the resource to help plan their menus.

### Sample size calculation

Based on estimates from a previous study, assuming a standard deviation of 0.5, a sample size of 78 (39 intervention and 39 control) would allow the detection of a difference in mean score of 0.3 in intentions to use, with 80 % power [13]. For serves of fruit and vegetables, assuming a standard deviation of 1, 78 services would allow the detection of 0.45 differences between intervention and control services, with 80 % power. As no prior knowledge was available regarding a meaningful effect of education interventions in childcare centres, the effect sizes was based on a consensus between study investigators.

### Analysis

Analysis was undertaken using STATA 11.0. Services with postcodes ranked in the top 50 % were categorised as ‘higher socioeconomic status’, and those grouped in the lower 50 % were categorised as ‘lower socioeconomic status’ (SES) using the 2011 Socio-Economic Indexes For Australia (SEIFA) scores [19]. This was compared between consenters and non-consenters using Pearson’s Chi-squared test. Descriptive statistics were generated for all responses and compared between intervention and control groups using appropriate univariate tests. Mean scores for each TPB construct were generated and compared between groups using student’s *t* test. A two-sided significance value of 0.05 was employed. Mean scores in intentions to use and serves of fruit and vegetable were also reported by whether services received the resource as well as by duration receiving the resource (four or more weeks ago or less than 4 weeks).

## Results

Of the 220 services, 106 were ineligible for the following reasons: menu planning did not occur onsite (*n* = 85, 31.7 %); cooks did not understand English (*n* = 16, 15.1 %); and others (*n =* 5, 4.7 %). Thirty-four services did not consent, 14 could not be contacted and 77 (68 % of eligible) (39 controls and 38 intervention) consented. There was no difference in SES between services that consented and did not consent to participate (44 % lower SES for consenters; 31 % lower SES for non-consenters; *p* value 0.1857). There were also no significant differences in cook and service characteristics between the groups (Table 1).

**Table 1:** Cook and service characteristics for those in the intervention and control group

Demographics	Intervention (*n* = 38)	Control (*n* = 39)	*p* value
Cooks’ characteristics			
Cooks age (years) mean (sd)	47.4 (10.3)	68.9 (15.3)	0.3919
Time as service cook (years) mean (sd)	10.1 (7.1)	7.2 (5.5)	0.0534
Sex *n* (%F)	37 (97)	38 (97)	0.985
Qualifications in nutrition			
TAFE course *n* (%)	14 (36.8)	10 (25.6)	0.289
Registered training course *n* (%)	13 (34.2)	15 (38.5)	0.698
Commercial cooking qualifications *n* (%)	5 (13.2)	8 (23.1)	0.259
Service characteristics			
Service sociodemographic			
High SES *n* (%)	20 (51)	23 (61.5)	0.361
Number of children catered for *n* (sd)	40.8 (17.3)	60.1 (13.6)	0.2582

### Intervention receipt

Overall, 32 (85 %) cooks in the intervention arm reported receiving the material, with time between receiving resource and participation in the follow-up assessment ranging from 1 to 8 weeks. Of those who reported receiving the resource, 19 (59 %) used the resource to plan their menu and 13 (41 %) reported that they were intending to use the resource.

### Trial outcomes

Cooks in the intervention arm had significantly higher mean scores on intentions to use the guidelines (*p* value 0.0005), perceived behavioural control (*p* value 0.0008) and attitudes (*p* value 0.0071) but not for social norm (*p* value 0.6088) (Table 2) compared to the control group. No significant differences were observed in serves of fruit and vegetables on the menu (Table 2). Mean scores for intentions to use and number of serves of fruit and vegetables on menus were also reported by whether services received the resource and length of time since receipt of the resource (see Table 3).

**Table 2:** Mean scores of the Theory of Planned Behaviour constructs and fruit and vegetable serves on menus by intervention group

	Intervention (*n* = 38)		Control (*n* = 39)		
Primary outcomes	Mean	sd	Mean	sd	*p* value
Intention^a,b^	6.6	0.4	6.2	0.6	0.0005*
Vegetable (number of serves)	3.8	1.1	3.3	0.8	0.0573
Fruit (number of serves)	2.9	0.6	2.9	0.7	0.7278
Secondary outcomes (TPB)					
Attitudes^a,b^	6.7	0.4	6.4	0.5	0.0071*
Subjective norm^a,b^	6.1	0.8	6.1	0.5	0.6088
Perceived behavioural control^a,b^	6.3	0.6	5.8	0.8	0.0008*

*Denotes *p* value <0.05

^a^Missing values for two services

^b^Constructs are reported on seven-point Likert scale ranging from 1 (strongly disagree) to 7 (strongly agree)

**Table 3:** Mean scores of the Theory of Planned Behaviour constructs and fruit and vegetable serves on menus by reported receipt of the resource and time since receiving the resource

	Intentions^b^		Serves of fruit		Serves of vegetables	
Reported receiving the resource^a^	Mean	sd	Mean	sd	Mean	sd
Yes (*n* = 32)	6.6	0.4	3.4	0.6	3.2	0.8
No (*n* = 3)	3.8	1.1	2.8	1.2	3.3	1.0
Time since receiving the resource						
≤4 weeks (*n =* 17)	6.2	0.6	2.6	0.6	3.5	0.8
>4 weeks (*n =* 15)	6.8	0.1	3.0	0.8	2.9	0.8

^a^Missing value for one service

^b^Intentions is reported on seven-point Likert scale ranging from 1 (strongly disagree) to 7 (strongly agree)

### Awareness of the guidelines and other implementation support

Seventy-nine percent of the control group and 84 % of the intervention group received a printed version of the Caring for Children resource (*p* value 0.570); and 51 % of the control group and 47 % of the intervention group received other support to implement the guidelines (*p* value 0.536). Of those receiving support, 61 % had attended a training workshop, 43 % participated in cooks network meetings and 57 % received face-to-face support from their local health promotion units.

## Discussion

In contrast to a previous trial undertaken in primary care [13], cooks who had received a printed educational resource had significantly higher intentions to use nutritional guidelines, together with perceived behavioural control and attitudes, compared to the control group. No difference in number of serves of fruit and vegetables on menus were observed suggesting that inclusion of education resources as part of multi-component interventions are likely required to modify food provision in care. This finding is consistent with reviews of trials undertaken in the health care setting which report small, but non-significant improvements to provider behaviour [15]. Findings in relation to provision of vegetables (*p* value 0.0573) appear promising and warrant further investigation using trials powered to detect small but meaningful changes in food provision.

The strengths of the study include its randomised design, blinded outcome assessment and theory-informed intervention development and evaluation. A one-item question was used to assess the provision of fruit and vegetables on menus. This measure is not validated and is likely to result in an overestimation of effect. This study only collected post-intervention data, which limits the ability to assess whether the intervention had differential effects on particular baseline characteristics. The study also had a short follow-up period of up to 2 months. The variation in time (1 to 8 weeks) between receiving the resource and when cooks undertook post-intervention data collection may have impacted on study findings as cooks had variable time to implement changes to their menus.

This study found that the use of printed educational materials can improve childcare service cooks’ intentions to use nutritional guidelines when planning their menus. Others strategies to support implementation however may be required to improve food provision in care.

## Additional files

Additional file 1: Application of the Theory of Planned Behaviour to development of intervention resource. (DOCX 21 kb)

Additional file 2: Twenty-one-item questionnaire used to assess the constructs from the Theory of Planned Behaviour. (DOC 485 kb)
