# Supplementary material for: A theory-based evaluation of a dissemination intervention to improve childcare cooks’ intentions to implement nutritional guidelines on their menus
Source: Implement Sci. 2016 Jul 25;11:105. doi: 10.1186/s13012-016-0474-7 (PMC4960853; doi:10.1186/s13012-016-0474-7)
Supplement: Supplementary file 1 — Application of the Theory of Planned Behaviour to development of intervention resource. (DOCX 21 kb) [file 13012_2016_474_MOESM1_ESM.docx]

**Additional file 1.** Application of the Theory of Planned Behaviour to development of intervention resource

| **Cooks belief^a^** | **Strategy** | **Application** |
| --- | --- | --- |
| “It is not possible to meet recommendations” | Establishing a new belief | An example of a sample menu that was compliant with guidelines was included on the menu. |
| “Children will not eat the new foods” | Acknowledge the belief and establish a new belief | The resource acknowledged these perceptions and highlighted that it could take children a little while to get used to these foods, and that initial rejection does not necessarily represent dislike of the food. |
| “There will be high wastage of fruit and vegetables” | Attack the strength of the behaviour | The resource highlighted case studies where waste did not increase when providing healthier foods.  The resource reinforced strategies that cooks/service could employ to overcome the barrier. |
| “Educators are not supportive” | Support ways of obtaining immediate benefits | The resource suggested that cooks engage with educators and service supervisors to ask for positive feedback. |

^a^ Cooks belief identified via research team interview with childcare centre cooks
