# Supplementary material for: A theory-based evaluation of a dissemination intervention to improve childcare cooks’ intentions to implement nutritional guidelines on their menus
Source: Implement Sci. 2016 Jul 25;11:105. doi: 10.1186/s13012-016-0474-7 (PMC4960853; doi:10.1186/s13012-016-0474-7)
Supplement: Supplementary file 2 — Twenty-one-item questionnaire used to assess the constructs from the Theory of Planned Behaviour. (DOC 485 kb) [file 13012_2016_474_MOESM2_ESM.doc]

**Additional file 2.** 21-item questionnaire used to assess the constructs from the Theory of Planned Behaviour

<Introductory text, demographic questions and non relevant text removed>

How many wholegrain/wholemeal/high fibre breads & cereals yesterday

*******************MULTIPLE CHOICE - CATI VERSION**************************

INFO 1 CHILDGU18 NOLAB

MODULE SUBMODUL

Nomt gt '0000000000'

In the next section we will ask you a series of questions about the

menu planning process you undertake, at each menu review,

to plan a menu that meets the childcare sector nutrition guidelines,

for the children who attend your service.

The childcare sector nutrition guidelines are outlined in the

‘Caring for Children’ resource which was first released in 2005.

The guidelines support early childhood education and care services with

menu planning to provide healthy foods to children attending care.

******************* INFORMATION SCREEN ITEM *******************************

INFO 1 CHILDGU28 NOLAB

CC Guidelines

CHILDGU1=1

The Caring for Children resource has recently been updated and

re-released in 2014 by the NSW Ministry of Health.

The guidelines are practical and based on the Australian Guide to

Healthy Eating.

You may have recently received a copy of the manual from your

local health district.

It is a ring bound yellow resource with four pictures on the front cover.

******************* INFORMATION SCREEN ITEM *******************************

CHCE 1 4 HCCG 1 _MAKE_ LABEL

CC Guidelines

CHILDGU2=1

Have you heard of the Caring for Children guidelines?

1 Yes

2 No

3 Don't Know

.R Refused

Heard Caring for Children guidelines

***************** SINGLE CHOICE - CATI VERSION ****************************

CHCE 1 4 Rec_CC 1 _MAKE_ LABEL

CC Guidelines

Have you received a copy of the Caring for Children manual?

1 Yes

2 No

3 Don't Know

.R Refused

Received CC guidelines

***************** SINGLE CHOICE - CATI VERSION ****************************

CHCE 1 4 Sup1 4 _MAKE_ LABEL

CC Guidelines

Rec_CC =1

Have you received any support to use the Caring for Children resource to

plan your menu in the last 6 months?

Prompt: For example, from your Local Health District.

1 Yes

2 No

3 Don't Know

.R Refused

Received support to use CC guidelines last 6 months

***************** SINGLE CHOICE - CATI VERSION ****************************

MULT 1 9 Sup2 8 7 mltlb

CC Guidelines

Sup1 = 1

If yes, what support have you received?

(Please select all that apply)

[INTERVIEWER NOTE: 'w'=with, 'info'=information, '&'=and]

1 Attended workshops

2 Participated in cooks networks

3 Received face to face support

4 Received telephone support

5 Received email support

6 Received printed resources or newsletters w info & tips

7 Other (please specify)

-8 I don't know [Do not read out]

.R Refused [Do not read out]

Support received

Attended workshops

Participated in cooks networks

Received face to face support

Received telephone support

Received email support

Received printed resources or newsletters with information and tips

Other (please specify)

I don't know [Do not read out]

Refused [Do not read out]

*******************MULTIPLE CHOICE - CATI VERSION**************************

OPEN 1 200 Sup2_oth1 LABEL

MODULE SUBMODUL

Sup2 gt '000000000' and substr(sup2,7,1)='1'

What other support have you received?

Other support received

******************* OPEN ENDED ENTRY ITEM *********************************

NULL 2 nulla 0 NOLAB

MODULE SUBMODUL

Sup2_oth gt '' or Sup2 gt '000000000' and substr(sup2,7,1)='0'

or sup1 in (2,3,.R)

*************************NULL ITEM - DOES NOTHING*************************

INFO 1 AWGU1 8 NOLAB

Caring for Children guidelines

nulla=1

We are aware that some of the questions we will be asking today might

seem repetitive.

This is to allow us to capture a broad range of factors that may be

relevant to your views on applying the Caring for children guidelines to

your service specifically.

Your answers will be extremely useful in helping us design resources to

best support childcare services with providing healthy foods for children.

******************* INFORMATION SCREEN ITEM *******************************

INFO 1 AWGU2 9 NOLAB

Caring for Children guidelines

AWGU1=1

I will now read out a list of statements about the program and ask you

to answer them on a scale of Strongly Disagree, Disagree,

Slightly disagree, Neither agree nor disagree, Slightly agree, Agree,

Strongly Agree

Again we remind you that the statements we are asking today relate to the

menu planning process you undertake, at each menu review,

to plan a menu that meets the childcare sector nutrition guidelines,

for the children who attend your service.

******************* INFORMATION SCREEN ITEM *******************************

CHCE 1 8 CGU200 2 _MAKE_ LABEL

CC Guidelines

CGUnew gt .

For me, it is good practice to plan a menu according to the

Caring for Children guidelines.

1 Strongly Disagree

2 Disagree

3 Slightly disagree

4 Neither agree nor disagree

5 Slightly agree

6 Agree

7 Strongly Agree

.R Refused

Considered good practice to use the CC Guidelines

***************** SINGLE CHOICE - CATI VERSION ****************************

CHCE 1 8 CGU35 2 _MAKE_ LABEL

CC Guidelines

CGU68 gt .

I INTEND to plan a menu according to the Caring for Children guidelines

in the NEXT SIX MONTHS.

1 Strongly Disagree

2 Disagree

3 Slightly disagree

4 Neither agree nor disagree

5 Slightly agree

6 Agree

7 Strongly Agree

.R Refused

INTEND Next 6m to plan a menu CC Guidelines

CHCE 1 8 CGU203 2 _MAKE_ LABEL

CC Guidelines

CGU6 gt .

For me, it is necessary to plan a menu according to the

Caring for Children guidelines

1 Strongly Disagree

2 Disagree

3 Slightly disagree

4 Neither agree nor disagree

5 Slightly agree

6 Agree

7 Strongly Agree

.R Refused

Necessary to plan menu according to CC Guidelines

***************** SINGLE CHOICE - CATI VERSION ****************************

CHCE 1 8 CGU54 2 _MAKE_ LABEL

CC Guidelines

CGU203 gt .

People who are important to me think that I should plan a menu according

to the Caring for Children guidelines.

1 Strongly Disagree

2 Disagree

3 Slightly disagree

4 Neither agree nor disagree

5 Slightly agree

6 Agree

7 Strongly Agree

.R Refused

plan a menu according CC Guidelines

***************** SINGLE CHOICE - CATI VERSION ****************************

CHCE 1 8 CGU56 5 _MAKE_ LABEL

CC Guidelines

CGU54 gt .

Other professionals also Plan a menu according to the Caring for

Children guidelines at every menu review.

Prompt:

Other professionals refers to ‘other Childcare service cooks’(anywhere)

1 Strongly Disagree

2 Disagree

3 Slightly disagree

4 Neither agree nor disagree

5 Slightly agree

6 Agree

7 Strongly Agree

.R Refused

Other professionals CC Guidelines

***************** SINGLE CHOICE - CATI VERSION ****************************

CHCE 1 8 CGU33 2 _MAKE_ LABEL

CC Guidelines

CGU56 gt .

I INTEND to plan a menu according to the Caring for Children guidelines

at every menu review.

1 Strongly Disagree

2 Disagree

3 Slightly disagree

4 Neither agree nor disagree

5 Slightly agree

6 Agree

7 Strongly Agree

.R Refused

INTEND to plan a menu CC Guidelines

***************** SINGLE CHOICE - CATI VERSION ****************************

CHCE 1 8 CGU57 3 _MAKE_ LABEL

CC Guidelines

CGU1 gt .

I can count on support from colleagues whom I work with

WHEN THINGS GET TOUGH Planning a menu according to the Caring for

Children guidelines at every menu review.

1 Strongly Disagree

2 Disagree

3 Slightly disagree

4 Neither agree nor disagree

5 Slightly agree

6 Agree

7 Strongly Agree

.R Refused

WHEN THINGS GET TOUGH CC Guidelines

***************** SINGLE CHOICE - CATI VERSION ****************************

CHCE 1 8 CGU58 3 _MAKE_ LABEL

CC Guidelines

CGU5 gt .

COLLEAGUES whom I work with are WILLING TO LISTEN TO MY PROBLEMS

when Planning a menu according to the Caring for Children guidelines

at every menu review.

1 Strongly Disagree

2 Disagree

3 Slightly disagree

4 Neither agree nor disagree

5 Slightly agree

6 Agree

7 Strongly Agree

.R Refused

WILLING TO LISTEN TO MY PROBLEM CC Guidelines

***************** SINGLE CHOICE - CATI VERSION ****************************

CHCE 1 8 CGU204 2 _MAKE_ LABEL

CC Guidelines

CGU7 gt .

For me, it is SATISFYING to plan a menu according to the

Caring for Children guidelines

1 Strongly Disagree

2 Disagree

3 Slightly disagree

4 Neither agree nor disagree

5 Slightly agree

6 Agree

7 Strongly Agree

.R Refused

It is satisfying to plan menu according to CC Guidelines

***************** SINGLE CHOICE - CATI VERSION ****************************

CHCE 1 8 CGU12 2 _MAKE_ LABEL

CC Guidelines

CGU19 gt .

I am CONFIDENT that I can plan a menu according to the

Caring for Children guidelines

1 Strongly Disagree

2 Disagree

3 Slightly disagree

4 Neither agree nor disagree

5 Slightly agree

6 Agree

7 Strongly Agree

.R Refused

CONFIDENT that I can plan CC Guidelines

***************** SINGLE CHOICE - CATI VERSION ****************************

CHCE 1 8 CGU201 2 _MAKE_ LABEL

CC Guidelines

CGU12 gt .

For me, it is HELPFUL to plan a menu according to the

Caring for Children guidelines

1 Strongly Disagree

2 Disagree

3 Slightly disagree

4 Neither agree nor disagree

5 Slightly agree

6 Agree

7 Strongly Agree

.R Refused

It is helpful to plan menu according to CC Guidelines

***************** SINGLE CHOICE - CATI VERSION ****************************

CHCE 1 8 CGU202 2 _MAKE_ LABEL

CC Guidelines

CGU201 gt .

For me, it is APPROPRIATE to plan a menu according to the

Caring for Children guidelines

1 Strongly Disagree

2 Disagree

3 Slightly disagree

4 Neither agree nor disagree

5 Slightly agree

6 Agree

7 Strongly Agree

.R Refused

It is appropriate to plan menu according to CC Guidelines

***************** SINGLE CHOICE - CATI VERSION ****************************

CHCE 1 8 CGU14 7 _MAKE_ LABEL

CC Guidelines

CGU74 gt .

I have the confidence to Plan a menu according to the Caring for

Children guidelines even when other professionals I work with are not

doing this.

PROMPT:

other professionals

refers to the educators that work within the childcare centre

1 Strongly Disagree

2 Disagree

3 Slightly disagree

4 Neither agree nor disagree

5 Slightly agree

6 Agree

7 Strongly Agree

.R Refused

other professionals CC Guidelines

***************** SINGLE CHOICE - CATI VERSION ****************************

CHCE 1 8 CGU15 3 _MAKE_ LABEL

CC Guidelines

CGU73 gt .

I have the confidence to plan a menu according to the Caring for

Children guidelines even when the CHILDREN who attend the service

are not RECEPTIVE

1 Strongly Disagree

2 Disagree

3 Slightly disagree

4 Neither agree nor disagree

5 Slightly agree

6 Agree

7 Strongly Agree

.R Refused

RECEPTIVE Children CC Guidelines

***************** SINGLE CHOICE - CATI VERSION ****************************

CHCE 1 8 CGU17 2 _MAKE_ LABEL

CC Guidelines

CGU8 gt .

For me, planning a menu according to the Caring for Children guidelines,

is EASY

1 Strongly Disagree

2 Disagree

3 Slightly disagree

4 Neither agree nor disagree

5 Slightly agree

6 Agree

7 Strongly Agree

.R Refused

Planning CC Guidelines EASY

***************** SINGLE CHOICE - CATI VERSION ****************************

CHCE 1 8 CGU36 2 _MAKE_ LABEL

CC Guidelines

CGU71 gt .

I have a STRONG INTENTION to plan a menu according to the Caring for

Children guidelines, at every menu review.

1 Strongly Disagree

2 Disagree

3 Slightly disagree

4 Neither agree nor disagree

5 Slightly agree

6 Agree

7 Strongly Agree

.R Refused

STRONG INTENTION to plan a menu CC Guidelines

***************** SINGLE CHOICE - CATI VERSION ****************************

CHCE 1 8 CGU59 2 _MAKE_ LABEL

CC Guidelines

CGU36 gt .

CHILDREN at the organisation where I plan a menu according to the

Caring for Children guidelines are RECEPTIVE.

1 Strongly Disagree

2 Disagree

3 Slightly disagree

4 Neither agree nor disagree

5 Slightly agree

6 Agree

7 Strongly Agree

.R Refused

RECEPTIVE CC Guidelines

***************** SINGLE CHOICE - CATI VERSION ****************************

CHCE 1 8 CGU55 2 _MAKE_ LABEL

CC Guidelines

CGU59 gt .

People WHOSE OPINION I VALUE would approve of me planning a menu

according to the Caring for Children guidelines at every menu review.

1 Strongly Disagree

2 Disagree

3 Slightly disagree

4 Neither agree nor disagree

5 Slightly agree

6 Agree

7 Strongly Agree

.R Refused

WHOSE OPINION I VALUE CC Guidelines

***************** SINGLE CHOICE - CATI VERSION ****************************

CHCE 1 8 CGU16 2 _MAKE_ LABEL

CC Guidelines

CGU20 gt .

I have PERSONAL CONTROL over planning a menu according to the

Caring for Children guidelines

1 Strongly Disagree

2 Disagree

3 Slightly disagree

4 Neither agree nor disagree

5 Slightly agree

6 Agree

7 Strongly Agree

.R Refused

PERSONAL CONTROL CC Guidelines

***************** SINGLE CHOICE - CATI VERSION ****************************

CHCE 1 8 CGU13 2 _MAKE_ LABEL

CC Guidelines

CGU72 gt .

I am CAPABLE of planning a menu according to the

Caring for Children guidelines, even when LITTLE TIME is available

1 Strongly Disagree

2 Disagree

3 Slightly disagree

4 Neither agree nor disagree

5 Slightly agree

6 Agree

7 Strongly Agree

.R Refused

CAPABLE of planning a menu CC Guidelines

***************** SINGLE CHOICE - CATI VERSION ****************************

CHCE 1 8 CGU34 2 _MAKE_ LABEL

CC Guidelines

CGU13 gt .

I WILL DEFINITELY plan a menu according to the Caring for Children

guidelines, at every menu review.

1 Strongly Disagree

2 Disagree

3 Slightly disagree

4 Neither agree nor disagree

5 Slightly agree

6 Agree

7 Strongly Agree

.R Refused

DEFINITELY to plan a menu CC Guidelines

<closing text removed>
